# Nedd4-1 is an exceptional prognostic biomarker for gastric cardia adenocarcinoma and functionally associated with metastasis

**DOI:** 10.1186/1476-4598-13-248

**Published:** 2014-11-14

**Authors:** Aiqin Sun, Guanzhen Yu, Xiaoyan Dou, Xiaowei Yan, Wannian Yang, Qiong Lin

**Affiliations:** School of Medical Sciences and Laboratory Medicine, Jiangsu University, Zhenjiang, 212013 China; Department of Oncology, Changzheng Hospital, Shanghai, China; Center for Health Research, Danville, PA 17822 USA; Weis Center for Research, Geisinger Clinic, Danville, PA 17822 USA

**Keywords:** Gastric cardia adenocarcinoma, Nedd4-1, Prognostic biomarker, Survival prediction

## Abstract

**Background:**

Gastric cardia adenocarcinoma (GCA) is the most aggressive subtype of gastric carcinoma. New molecular markers and therapeutic targets are needed for diagnosis, prognosis and treatment of GCA. This study is to establish the E3 ubiquitin ligase Nedd4-1 as a prognostic biomarker to predict the survival and guide the treatment of GCA patients.

**Methods:**

Expression of Nedd4-1 in 214 GCA tumor samples was detected by immunohistochemistry staining (IHC) using tissue microarray assay (TMA). Association of Nedd4-1 with cumulative survival of the TNM stages I-III patients and clinicopathological characteristics was statistically analyzed. The role of Nedd4-1 in gastric cancer cell migration and invasion were determined by transwell and wound healing assays.

**Results:**

Nedd4-1 is overexpressed in 83% of the GCA tumors. The 5-year survival rate in Nedd4-1 negative GCA patients is as high as 96%. Log-rank analysis indicated that overexpression of Nedd4-1 is inversely correlated with cumulative survival (*χ*^2^ = 21.885, *p* <0.001). Multivariate logistic regression analysis showed that overexpression of Nedd4-1 is associated with an extremely low GCA survival rate with a hazard ratio (HR) = 0.068 (*p* = 0.008) in TNM stages I-III patients. Statistical analysis of association of Nedd4-1 overexpression with clinicopathological characteristics revealed that overexpression of Nedd4-1 is tightly associated with TNM stage (*p* < 0.001). Knockdown of Nedd4-1 in gastric cancer cell lines AGS and N87 dramatically inhibited the gastric cancer cell migration and invasion.

**Conclusions:**

Our results indicate that Nedd4-1 is an exceptional prognostic biomarker for GCA and suggest that Nedd4-1 may play an essential role in GCA metastasis.

**Electronic supplementary material:**

The online version of this article (doi:10.1186/1476-4598-13-248) contains supplementary material, which is available to authorized users.

## Background

Gastric cancer was the fifth most common cancer and one of the highest mortality cancer types. Gastric cardia adenocarcinoma (GCA), a sub-type of adenocarcinoma of esophagogastric junction (AEJ) localized about 2 centimeters below esophagogastric junction (gastric cardia), is the most aggressive type of gastric carcinoma [1–3]. Incidents of GCA in Asia have been increasing in recent years. Currently, surgery is still the major therapeutic means for GCA patients. There has been no effective post-surgery treatment for GCA. Metastasis and post-surgery recurrence rates in GCA are as high as 40-65% [4]. How to effectively treat GCA is a big challenge faced in gastric cancer therapy.

Metastasis and post-surgery recurrence are the major causes for poor prognosis in GCA. Thus, treatment of metastasized and post-surgery GCA patients is crucial for improving therapeutic outcome and increasing GCA survival rate. There are a few molecular biomarkers of GCA, such as Sirt1, ErbB2, MCM2, identified in recent years [5–7]. Very recently, Nedd9, a Crk-associated protein involved in focal adhesion, has been found overexpressed in gastric cancer [8–10]. Expression of Nedd9 is tightly associated with gastric cancer progression, particularly metastasis. Thus, Nedd9 is proposed as a prognostic biomarker for gastric cancer. However, expression of Nedd9 in GCA is not known. Currently, none of the biomarkers has been used for diagnosis and prognosis or as therapeutic targets for GCA, or guiding the post-surgery treatment and targeted therapy. The EGFR inhibitor Erlotinib was applied for, but failed in phase II clinical trial of the targeted therapy in GCA [11]. Molecular events of carcinogenesis and metastasis in GCA are still poorly understood. New molecular markers and therapeutic targets are needed for diagnosis, prognosis and treatment of GCA.

Nedd4-1 is a member of the HECT domain-containing E3 ubiquitin ligase family. The cellular function of Nedd4 was initially found in regulation of degradation of epithelial sodium channel (ENaC) [12]. Defect in ubiquitination of ENaC by Nedd4 causes hypertension named Liddle Syndrome [13]. Nedd4-1 is involved in regulation of many intracellular signaling molecules, endocytic or vesicle sorting proteins, such as Cbl, Eps15, Tsg101, Hrs, SCAMPs, and ACK1 [14–20]. Knockdown of Nedd4-1 in A549 cells inhibited ligand-induced degradation of EGFR and significantly elevated expression level of EGFR [20].

The role of Nedd4-1 in tumorigenesis has been brought to attention in recent years. Nedd4-1 has been shown to interact with, ubiquitinate and down-regulate the tumor suppressor pTEN, and proposed as a pro-oncogenic protein [21]. Immunohistochemical (IHC) staining studies observed that Nedd4-1 is overexpressed in lung, colon and breast tumors [22–24]. These studies suggest that Nedd4-1 may play an important role in carcinogenesis. Expression of Nedd4-1 in gastric tumor has been investigated, however, the results were inconsistent [25, 26]. In addition, no correlation of Nedd4-1 expression with clinicopathological categories has been found in gastric cancer [26]. Down-regulation of the tumor suppressor pTEN is in general thought to be the molecular mechanism underlying Nedd4-1-associated oncogenesis [22–24]. However, there is still a debate on pTEN as an ubiquitination substrate of Nedd4-1 and a cause for Nedd4-1-associated oncogenesis [27–29]. A recent study found that Nedd4-1 down-regulates Lats1, a serine/threonine kinase in the Hippo pathway, suggesting that Nedd4-1 may activate YAP/TAZ signaling for promoting oncogenesis [30].

Given that Nedd4-1 is an important pro-oncogenic protein and little is known about its functional association with clinicopathological characteristics of tumors, we attempt to further define the role of Nedd4-1 in tumor genesis and progression at a clinical setting. In this report, we detected expression of Nedd4-1 in 214 GCA tumor samples with immunohistochemical (IHC) staining and found that Nedd4-1 overexpressed in 83% of GCA tumors. The Nedd4-1 negative GCA patients in TNM stage II/III had 100% of 5-year survival rate, suggesting that Nedd4-negative staining could be used for prediction of survival in GCA. On the other hand, overexpression of Nedd4-1 in GCA is inversely associated with survival and tightly correlated with metastasis of GCA. Furthermore, we have shown that depletion of Nedd4-1 by shRNA in gastric cancer cells impairs both the basal and EGF-stimulated cell migration and invasion, indicating that Nedd4-1 plays a “driver” role in metastasis of GCA. Our studies for the first time demonstrate that Nedd4-1 is an exceptional biomarker for prognosis of GCA. The functional association of Nedd4-1 with GCA metastasis suggests Nedd4-1 as a potential anti-metastatic drug target for GCA therapy.

## Results

### Clinicopathological information of the GCA tumor samples used for IHC staining of Nedd4-1

Total 214 GCA tumor samples were collected. The clinicopathological information is listed in Table 1. In this GCA patient population, the occurring frequency of GCA in or below and above age 60 is almost even (108 for < = 60 year-old, and 106 for >60 year-old). Roughly three quarters of the patients were male. More than three quarters of tumors were equal to or smaller than 6 centimeters. About two thirds of the patients were diagnosed with the tumors highly invaded (T3/T4) or metastasized to lymph nodes (N1-3 categories) or at advanced TNM stages (stage III/IV).

**Table 1:** **Pathological information of gastric cardia adenocarcinoma samples**

Pathological category		Case number	% of total cases
Age	< = 60	108	50.5
	>60	106	49.5
Gender	Male	157	73.4
	Female	57	26.6
Tumor size	< = 6 cm	166	77.6
	>6 cm	48	22.4
T category	T1/T2	63	29.4
	T3/T4	151	70.6
N category	N0	73	34.1
	N1-3	141	65.9
Differentiation	Well/Moderately	130	60.7
	Poorly/undiff	84	39.3
TNM-stage	I/II	83	38.8
	III/IV	131	61.2

**Figure 1:**
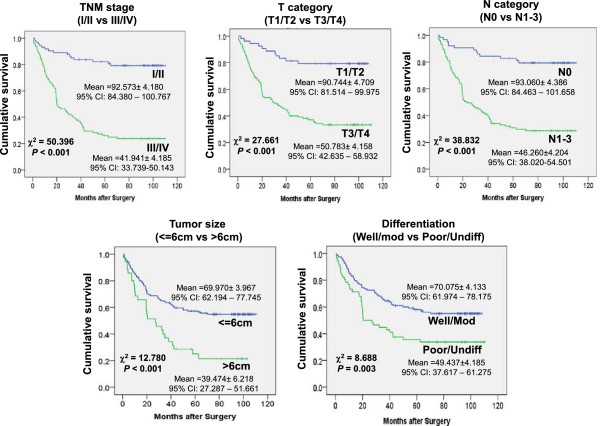
**Association of pathological characteristics with cumulative survival in GCA patients.** Association of tumor size, invasion (T category), lymph node metastasis (N category), differentiation, and TNM stages with cumulative survival of GCA patients is shown in Kaplan-Meier survival graphs. The Chi-square and *p* values from Mantel-Cox test and the mean survival for each pathological category are shown in the figure.

The post-surgery survival rate of the GCA patients was followed and censored. Overall mean survival time of the patients is 64.036 ± 3.554 months (95% confidential interval (CI): 57.070 – 71.002). Association of the pathological categories with cumulative survival is displayed by Kaplan-Meier survival graph and statistically analyzed by log-rank test (Figure 1). The mean survival time (in month) and 95% CI were calculated and also shown in Figure 1. The data have shown that differences between tumor sizes, invasive degrees (T categories), lympho node metastasis (N categories), grades (differentiation) and TNM (T category, N category and remote metastasis) stages are all significantly and inversely associated with patient survival (*p* = 0.003 or <0.001). The chi-square values from log-rank test indicate that difference in cumulative survival between the TNM-stages is the most significant, followed by tumor sizes and grades (Figure 1). In categories of TNM stage, N category is more significant than T category. These data suggest that tumor metastasis might be the major factor related to mortality of the GCA patients.

### Nedd4-1 is tightly associated with poor prognosis of gastric cardia adenocarcinoma

**Figure 2:**
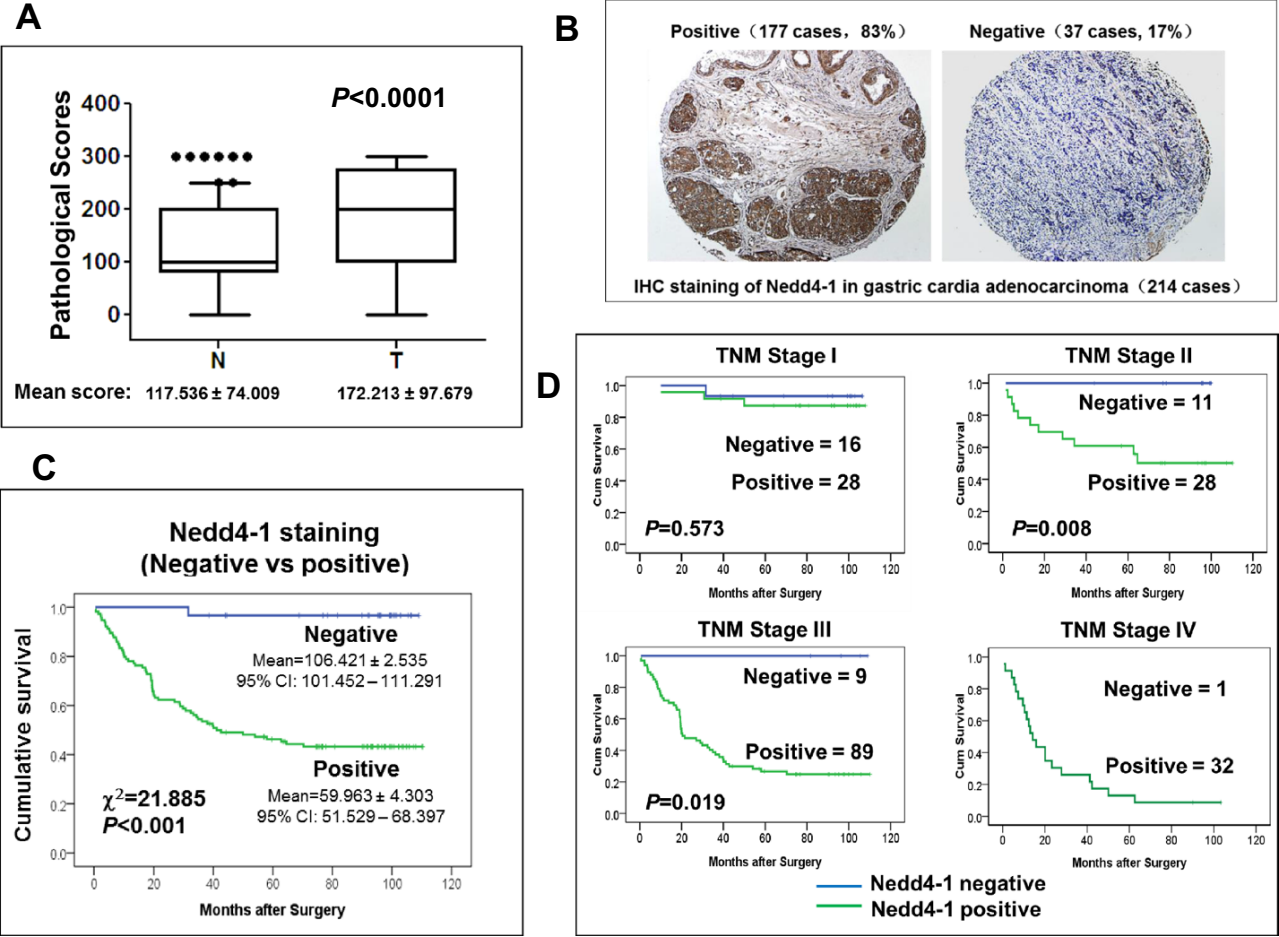
**Nedd4-1 is overexpressed in GCA and the overexpression is tightly negatively correlated with cumulative survival of GCA patients. A**, Overexpression of Nedd4-1 in GCA tumor tissue is significantly higher than in their adjacent normal gastric cardia tissue. The average scores and standard deviations of IHC staining of Nedd4-1 in both GCA tumors (T) and their adjacent normal tissue (N) are shown in the figure. **B**, IHC staining of Nedd4-1 in GCA tumor samples. **C**, Kaplan-Meier survival curve of Nedd4-1 positive and negative GCA patients in TNM stages I-III. The Chi-square and *p* values from Mantel-Cox test and the mean survival for Nedd4-1 negative and positive GCA of TNM stages I-III are shown in the figure. **D**. Kaplan-Meier survival curve of Nedd4-1 positive and negative GCA patients in each of TNM stages. The *p* value was calculated from Mantel-Cox test.

The E3 ubiquitin ligase Nedd4-1 has been identified as a pro-oncogenic protein and is overexpressed in lung, colon and breast cancers [20–23]. To detect Nedd4-1 in GCA, we raised an anti-Nedd4-1 antibody in rabbits using GST-human Nedd4-1 fusion protein as an antigen. As shown in the figures in Additional file 1, this anti-Nedd4-1 antibody recognizes both ectopically and endogenously expressed Nedd4-1 in immunoblotting and immunofluorescent staining, indicating that this anti-Nedd4-1 is highly specific and able to detect Nedd4-1 in both denaturing and non-denaturing conditions. The expression of Nedd4-1 in the 214 GCA tumors and their adjacent normal tissues was then examined by the TMA assay with IHC staining. As shown in Figure 2A, expression of Nedd4-1 in the GCA tumors is significant higher than in their adjacent normal tissues (*p* < 0.0001). The TMA assay has shown that 177 of the 214 GCA tumors (83%) were strongly stained with anti-Nedd4-1, and only 37 tumor samples (17%) had weak or no staining (Figure 2B), indicating that Nedd4-1 is overexpressed in GCA with a very high frequency.

To assess the role of Nedd4-1 in prognosis, we determined association of Nedd4-1 expression with cumulative survival of the GCA patients in TNM stages I-III (181 cases) by statistical analysis. As shown in the Kaplan-Meier survival graph (Figure 2C), the patients with the Nedd4-1 negative tumor had average cumulative survival of 106.421 ± 2.535 months during the follow-up. The 5 year survival rate in the Nedd4-1 negative GCA patients is estimated as high as 96%. On the other hand, the Nedd4-1 positive patients had average cumulative survival of 59.963 ± 4.303 months (Figure 2C). The 5 year survival rate in the Nedd4-1 positive patients is estimated as 45%, significantly lower than that of the Nedd4-1 negative patients. Difference between the Nedd4-1 positive and negative patient’s cumulative survival is determined by log-rank test (Figure 2C). The chi-square value is 21.885 with *p* < 0.001, indicating that cumulative survival between the Nedd4-1 positive and the negative patients is dramatically different and that Nedd4-1 expression is inversely associated with post-surgery survival in TNM stages I-III patients.

Association of Nedd4-1 expression with cumulative survival in each of TNM stages was further analyzed with Kaplan-Meier graphs (Figure 2D). The data have shown that overexpression of Nedd4-1was significantly inversely associated with cumulative survival in TNM stages II/III patients (*p* = 0.008 and 0.019 respectively), but not associated with the cumulative survival in TNM stage I GCA patients (*p* = 0.573). The graphs in Figure 2D clearly show that all the Nedd4-1 negative GCA patients in TNM stages II/III were survived throughout the follow-up, indicating that Nedd4-1 negative staining is an exceptional index for predicting survival of the GCA patients diagnosed in TNM stages II/III. Because Nedd4-1 expressed in almost all the collected tumors in TNM stage IV patients, the Nedd4-1 negative staining as a survival predictor cannot be evaluated in this group of GCA patients.

The association of cumulative survival with the pathological categories and expression of Nedd4-1 was further analyzed using multivariate Cox regression model (Table 2). The survival rate for Nedd4-1 overexpression is much lower than other pathological categories (among TNM stages I-IV patients, HR = 0.07 with 95% CI: 0.009-0.52; among TNM stages I-III patients, HR = 0.068 with 95% CI: 0.009-0.495). Furthermore, TNM stage and N category are significant factors inversely associated with the survival rate (HR = 0.617, 95% CI: 0.382-0.998 for TNM stage, and HR = 0.364, 95% CI: 0.15-0.88 for N category). The data clearly indicate that Nedd4-1 overexpression is the best predictor for poor prognosis of GCA, followed by N category or TNM stage. As N category and TNM stage are known to be positively correlated with metastasis, these data suggest that metastasis is the key process in poor survival of GCA.

**Table 2:** **Multivariate Cox regression analysis of correlation of clinicopathological characteristics and Nedd4-1 with AGC cumulative survival**

Category	Hazard ratio	***P***
	(95% CI)	
TNM stage	0.617 (0.382-0.998)	0.049
T category	0.513 (0.211-1.246)	0.140
N category	0.364 (0.150-0.882)	0.025
Differentiation	0.819 (0.521-1.288)	0.388
Tumor size	0.932 (0.317-2.739)	0.899
Nedd4-1^*a*^	0.070 (0.009-0.521)	0.009
Nedd4-1^*b*^	0.068 (0.009-0.495)	0.008

^*a*^TNM stage IV samples were included in the analysis.

^*b*^TNM stage IV samples were excluded in the analysis.

TNM stage IV samples were included in the analysis of all other pathological categories.

### Nedd4-1 is associated with metastasis of GCA

We further statistically analyzed correlation of Nedd4-1 overexpression with pathological characteristics in GCA tumors. As shown in Table 3, Nedd4-1 overexpression is tightly associated with TNM stages, N and T categories, but not with age (*p* = 0.631), gender (*p* = 0.448), tumor size (*p* = 0.319) or grade (differentiation) (*p* = 0.066). In T category, 88.7% of the T3/T4 GCA patients were Nedd4-1 positive, compared to 68.3% of the T1/T2 patients (*p* = 0.001). In N category, Nedd4-1 positive staining is observed in 90.1% of N1-3 GCA patients (spread to regional lymph nodes), versus 68.5% for no lymph node spreading (*p* <0.001). In TNM stages, Nedd4-1 positive staining is observed in 97% of stage IV patients who had distant site metastasis and 90.8% of stage III patients who had severe lymph node metastasis, compared to 63.6% of stage I patients and 71.8% of stage II patients who had no or minor lymph node metastasis. The data in Table 3 strongly suggest that Nedd4-1 plays an important role in GCA progression and metastasis.

**Table 3:** **Association of Nedd4-1 expression with pathological categories of gastric cardia tumors**

Pathological category		Case number	Nedd4-1 Positive	%	***p***
Age	< = 60	108	88	81.5	0.631
	>60	106	89	84	
Gender	Male	157	128	81.5	0.448
	Female	57	49	86	
Tumor size	<=6 cm	166	135	81.3	0.319
	>6 cm	48	42	87.5	
T category	T1/T2	63	43	68.3	0.001
	T3/T4	151	134	88.7	
N category	N0	73	50	68.5	< 0.001
	N1-3	141	127	90.1	
Differentiation^*a*^	Well/Mod	130	103	79.2	0.066
	Poor/Undiff	84	74	88.1	
TNM stage	I	44	28	63.6	
	II	39	28	71.8	I/II vs III/IV
	III	98	89	90.8	<0.001
	IV	33	32	97.0	

^*a*^In differentiation category, “Well” stands for well differentiated, “Mod” for moderately differentiated, “Poor” for poorly differentiated, “Undiff” for undifferentiated.

**Figure 3:**
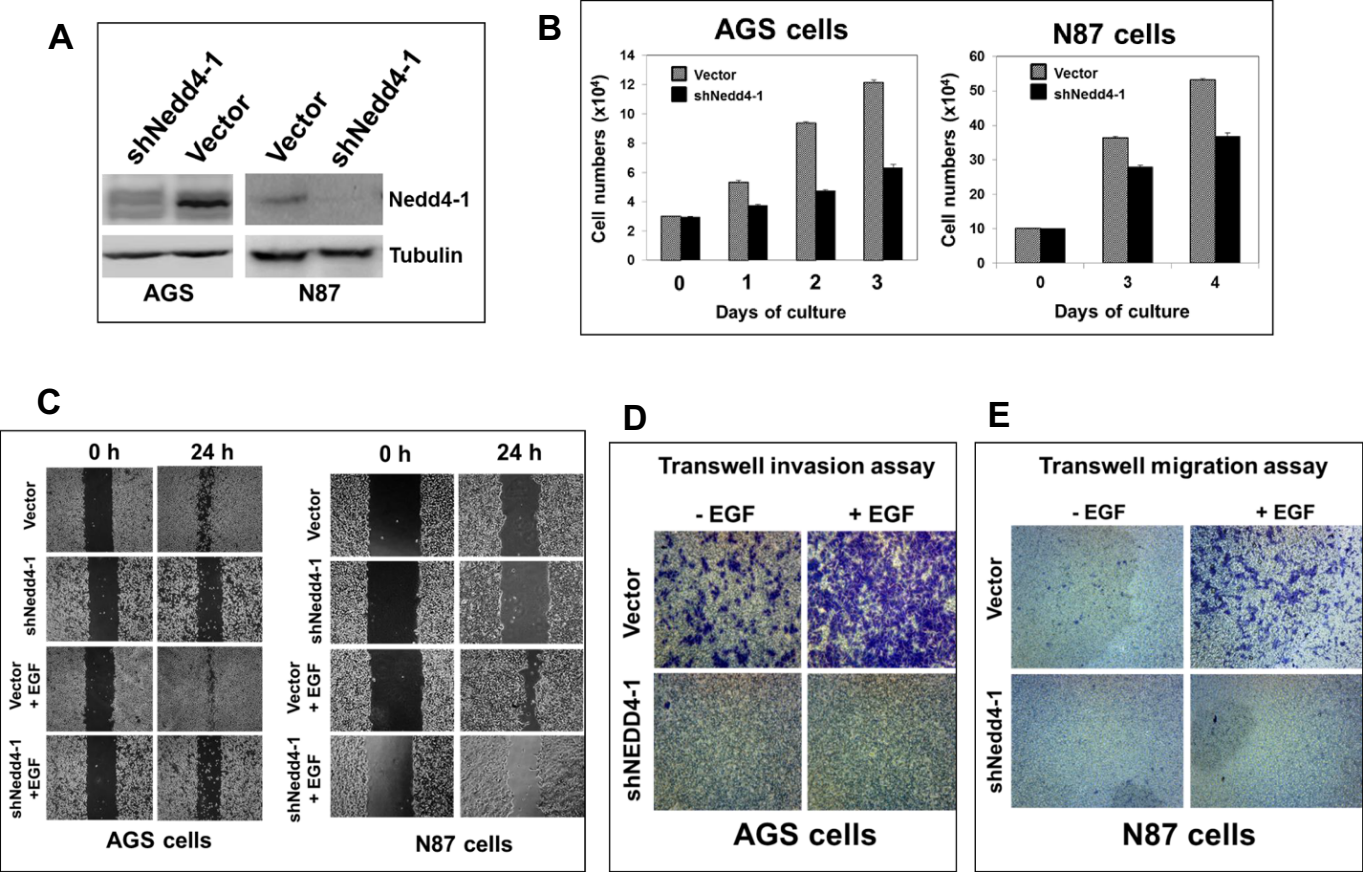
**Nedd4-1 is essential for gastric cancer cell invasion and migration. A**, Nedd4-1 in gastric cancer AGS and N87 cells was depleted by lentiviral vector-loaded Nedd4-1 shRNA. The gastric cancer cells were infected by lentiviral vector-loaded Nedd4-1 shRNA for 48 hours and endogenous Nedd4-1 in the cell lysates was detected by immunoblotting with anti-Nedd4-1. **B**, The effect of Nedd4-1 knockdown on AGS and N87 cell proliferation. **C**, The wound healing assay of the effect of Nedd4-1 knockdown on the gastric cancer cell migration. **D**, The invasion of AGS cells was determined by the transwell matrigel invasion assay. **E**, The migration of N87 cells was detected by the Transwell migration assay. The gastric cancer cell proliferation, migration and invasion assays were repeated three times and the results were consistent.

To verify the function of Nedd4-1 in GCA metastasis, we used two gastric cancer cell lines N87 and AGS [31, 32] to determine the role of Nedd4-1 in promoting cell migration and invasion, the two important cellular processes in metastasis. N87 was derived from the liver metastasis of a gastric cancer [31] and AGS was demonstrated to be metastatic [33], and both cell lines were used for cancer cell migration and invasion assays in previous studies [34, 35]. We first established the Nedd4-1-knockdown cell lines in both AGS and N87 using lentiviral vector-loaded Nedd4-1 shRNA. More than 90% of Nedd4-1 was depleted in the sh-Nedd4-1 cell lines (Figure 3A). Depletion of Nedd4-1 inhibited cell proliferation significantly in both AGS and N87 cells, but less severely in N87 cells (Figure 3B). We then determined effect of Nedd4-1 knockdown on cell migration and invasion in AGS cells and cell migration in N87 cells. As shown in Figure 3C, depletion of Nedd4-1 by the shRNA dramatically inhibited both basal and EGF-promoted cell migration in both cell lines using the wound healing assay. In the transwell invasion or migration assay, the basal and EGF-promoted cell invasion in AGS cells and migration in N87 cells were completely diminished by knockdown of Nedd4-1 (Figures 3D and E). These results indicate that Nedd4-1 promotes both basal and the EGF-promoted cell migration and invasion in gastric cancer cells. Taken together, our studies from the IHC staining of GCA tumor tissues and the cell invasion and migration assays of the Nedd4-1-depleted AGS and N87 cells suggest that Nedd4-1 is a crucial “driver” protein in metastasis of GCA that is the major cause for GCA mortality.

## Discussion

GCA is one of the most aggressive cancer types that affect millions of people’s life and living quality worldwide, particularly in Asia countries. Currently, no effective chemo or targeted therapeutic method has been established for GCA patients. Elucidation of pathological mechanisms and identification of diagnostic and prognostic biomarkers and therapeutic targets are urgent tasks for improving diagnosis and treatment of GCA. Here we identified the HECT E3 ubiquitin ligase Nedd4-1 as a predictive biomarker of poor prognosis of GCA. IHC staining of 214 GCA tumor samples has shown that Nedd4-1 was overexpressed in 83% of GCA tumors. Statistical analysis of the IHC staining data and the GCA tumor clinicopathological data found a very significant association of Nedd4-1 negative GCA with the post-surgery survival, especially in TNM stages II/III patients. The pathological data of the 214 GCA tumor samples suggest that metastasis is an important factor of poor prognosis. Analysis of association of Nedd4-1 overexpression with pathological categories has shown that Nedd4-1 overexpression is linked to GCA metastasis. The studies with knockdown of Nedd4-1 in gastric cancer AGS and N87 cells demonstrated that Nedd4-1 has a major role in promoting the gastric cancer cell invasion and migration, the two key cellular processes in metastasis, and an effect on the cell proliferation as well. Our work suggests that Nedd4-1, as a predictive biomarker of poor prognosis of GCA, functions in GCA progression, particularly in metastasis.

Nedd4-1 is an HECT E3 ubiquitin ligase that is known to ubiquitinate the tumor suppressor pTEN and causes proteasomal degradation and nuclear translocation of pTEN [21, 36]. This activity of Nedd4-1 was proposed as the pro-oncogenic role of Nedd4-1 [21, 36]. However, recent studies have raised questions about the role of Nedd4-1 in ubiquitination of pTEN for degradation and nuclear translocation [27, 28]. Nevertheless, overexpression of Nedd4-1 has been found in multiple types of tumors by IHC staining [22–24]. A recent report claimed that overexpression of Nedd4-1 in colon cancer tissues was not correlated with down-regulation of pTEN [29], suggesting that at least in some cases the function of Nedd4-1 in oncogenesis or tumor progression may not be dependent on down-regulation of pTEN. In our studies, the major role of Nedd4-1 seems promoting tumor cell migration and correlated to GCA metastasis. It is not clear whether Nedd4-1 promotes GCA metastasis through down-regulation of pTEN. Multiple substrates of Nedd4-1 in addition to pTEN have been identified. These substrates include receptor or non-receptor tyrosine kinases, serine/threonine kinases and other signaling molecules [14–20]. In addition, very recent studies found that the Crk-associated focal adhesion protein Nedd9 is overexpressed in gastric cancer and tightly associated with metastasis [8–10]. The functional resemblance of Nedd9 to Nedd4-1 in gastric cancer metastasis may be yielded from a connected biochemical process. Taken together, Nedd4-1 may promote GCA progression and metastasis through complex signaling pathways.

Two research groups have investigated overexpression of Nedd4-1 in gastric tumor tissues [25, 26]. One group observed no up-regulation of Nedd4-1 and no correlation with pTEN expression in 50 gastric cancer tumor samples [25]. The other found that Nedd4-1 was overexpressed in 75% of 60 gastric tumor samples, but expression of Nedd4-1 had no significant association with clinicopathologic characteristics, including invasion, metastasis and stage [26]. These results are inconsistent with our studies in GCA. The discrepancy may be produced from differences in gastric tumor types or tumor sample numbers. GCA is a very unique type of gastric cancer that has much higher mortality and poorer prognosis than general gastric cancer due to its high invasive and metastatic properties.

Our studies indicate that Nedd4-1 is an exceptional biomarker for predicting poor prognosis of the post-surgery GCA. Thus, detection of overexpression of Nedd4-1 by IHC in GCA tumors may be applied for GCA clinic and used to guide GCA treatment. The Nedd4-1 negative GCA patients should be treated differentially from the Nedd4-1 positive GCA patients. As the Nedd4-1 negative GCA patients in TNM stages II and III had as high as 100% of 5-year survival rate (Figure 2D), Nedd4-1 negative staining could be used for prediction of GCA patient’s post-surgery survival and guiding the post-surgery treatment. In addition, pathological classification of the GCA patients with Nedd4-1 IHC staining scores should be established upon further investigation with more cases, and used for diagnosis of GCA in combination with TNM-stage or T/N categories in clinic. Our studies also suggest that Nedd4-1 is a new target molecule for anti-cancer therapy of GCA as evidenced by its inverse association with GCA patient survival and essential role in the gastric cancer cell invasion and migration. Inhibition of Nedd4-1 may be an efficient approach for blocking GCA metastasis or relapse and improving survival rate of GCA patients.

Our data for the first time show that Nedd4-1 promotes gastric cancer cell invasion and migration and is tightly associated with GCA metastasis. In particular, Nedd4-1 mediates EGF-dependent gastric cancer cell invasion and migration, suggesting that Nedd4-1 may participate in EGFR-mediated tumor metastasis signaling. It is known that EGFR signaling plays an important role in cell migration and invasion in multiple types of cancer [37–41]. Mutations of EGFR have been identified as a key driving cause in tumorigenesis and progression of non-small cell lung carcinoma (NSCLC) [42–44]. How Nedd4-1 mediates EGFR cell migration and invasion signaling in both GCA and NSCLC cells is an important question for understanding the role of Nedd4-1 in tumor genesis and progression and needed to investigate further.

## Conclusions

The E3 ubiquitin ligase Nedd4-1 is overexpressed in 83% of GCA tumors. The expression of Nedd4-1 is inversely associated with cumulative survival of GCA patients. Nedd4-1 negative GCA patients had a striking high survival rate, suggesting that Nedd4-1 negative staining could be used for prediction of post-surgery survival of GCA. Overexpression of Nedd4-1 is tightly associated with TNM stage of GCA, and knockdown of Nedd4-1 in gastric cancer cells dramatically reduces the cell migration and invasion capability, indicating that Nedd4-1 is functionally associated with GCA metastasis. Our studies conclude that Nedd4-1 is an exceptional prognostic biomarker for prediction of post-surgery survival and a potential anti-metastatic target in GCA therapy.

## Methods

### Materials

Lentiviral human Nedd4-1 shRNA clones were purchased from Open Biosystems. The mAb against the hemagglutinin (HA) (12CA5) was purchased from Roche Applied Science. The polyclonal anti-Nedd4-1 antibody was made by injecting GST-human Nedd4-1 fusion protein into rabbits and purified by protein A beads. IHC staining S-P kit (KIT-9710) was purchased from MAIXIN Biology Corporation. The gastric cancer cell lines AGS and N87 were purchased from ATCC.

### Human tissue specimens and patient information

Tissue microarrays containing 214 cases with primary gastric cardia adenocarcinoma were used for detection of Nedd4-1 expression, which were preserved in the Gastric Cancer Tissue Bank at Department of Oncology, Changzheng Hospital (Shanghai, China). All the cases received curative resection. All of the tissue specimens for this study were obtained with patient informed consent, and the use of these GCA specimens was approved by the Changzheng and Changhai Hospital Institutional Review Board.

### Immunohistochemistry (IHC)

Standard procedure was performed to determine the level of Nedd4-1 expression in the GCA tumor samples. Briefly, 4 μm sections of paraffin-embedded GCA tissue microarrays were de-paraffinized and rehydrated in xylene and alcohol bath solution. Antigen unmasking was performed by pretreatment of the slides in 0.01 M citrate buffer (pH 6.0) at 98°C for 5 min using a microwave oven. The slides were then cooled to room temperature. Endogenous peroxidase was eliminated by incubating the slides in 3% hydrogen peroxide for 10 min. After washed in 10 mM PBS (pH 7.4), the sections were incubated with normal goat serum at room temperature for 10 min, followed by incubation with anti-Nedd4-1 antibody (dilution: 1:100) at 4°C overnight. An IHC staining S-P kit (KIT-9710; MAIXIN Biology Corporation, Fuzhou, China) was used to visualize antibody binding on the slides. Counterstaining was performed with hematoxylin. The IHC staining of Nedd4-1 in these specimens was evaluated by two individuals under an Olympus CX31 microscope (Olympus, Center Valley, PA).

### Evaluation of immunostaining

A mean percentage of Nedd4-1 positive tumour cells was determined in at least five areas at × 400 magnifications (50–250 cancer cells per area) and assigned from 0 to 100. The intensity of immunostaining was scored as follows: weak, 1+, moderate, 2+; and intense, 3+. Theoretically, the percentage of Nedd4-1 positive tumor cells and the staining intensity were multiplied to produce a weighted score for each case: ranging from 0 (0% of cells staining) to 300 (100% of the cells staining at 3+ intensity). For convenience in reporting and statistical analysis, the staining was classified as negative, weak, moderate and strong. The cut-off points were based on the scores: negative, 0; weak, <75; moderate, 75–150; and intense, >150. The score <75 is defined as low expression, and >75 as high expression.

### Cell culture and knockdown of Nedd4-1 by lentiviral vector-loaded shRNA in gastric cancer cells

Gastric cancer cell lines AGS and N87 was purchased from ATCC and maintained in Dulbecco's modified Eagle's medium (DMEM) (Hi-Clone) plus 10% FBS at 37°C with 5% CO_2_.

Lentiviral particle packaging was performed as following. Briefly, the lentiviral shRNA plasmid was co-transfected with psPAX2 (Addgene) and pMD2.G (Addgene) into HEK293KT cells for 8 hrs. The viral particle-containing medium was collected every 24 hours for three times after transfection. The medium was centrifuged at 1000xg for 5 min to remove cell debris, and used for infecting the gastric cancer cells in presence of 6 μg/ml polybrene. The infected gastric cancer cells were selected with puromycin, and the effect of Nedd4-1 knockdown was detected by immunoblotting the cell lysates with anti-Nedd4-1.

### Preparation of cell lysates and immunoblotting

Cells were rinsed once with ice-cold PBS and lysed in ice-cold Mammalian lysis buffer (40 mM Hepes (pH 7.4), 100 mM NaCl, 1% Triton X-100, 25 mM glycerol phosphate, 1 mM sodium orthovanadate, 1 mM EDTA, 10 μg/ml aprotinin, and 10 μg/ml leupeptin) or RIPA buffer (40 mM Hepes, pH 7.4, 1% Triton X-100, 0.5% Na-deoxylcholate, 0.1% SDS, 100 mM NaCl, 1 mM EDTA, 25 mM β-glycerolphosphate, 1 mM Na-orthovanadate, 10 μg/ml leupeptin and aprotinin) as indicated. The cell lysates were cleared by centrifugation at 13,000 rpm for 15 minutes. The cell lysates were denatured by addition of sample buffer and boiled for 5 minutes, resolved by 8%-14% SDS-PAGE, and then transferred to PVDF membranes (millopore). The membranes were incubated with primary antibodies 12 hrs at 4°C and HRP-conjugated second antibodies for 3–5 hrs at 25°C. The protein bands were detected by the chemiluminescence kit (Fuji).

### Determination of cell proliferation, migration and invasion of gastric cancer cells

*(1) Determination of cell proliferation.* The control or the Nedd4-1 knockdown N87 cells were cultured in DMEM with 10% FBS at 37°C plus 5% CO2 for indicated times. The cells were trypsinized and counted under a phase microscope with a hemocytometer. The cell counting was repeated at least three times.

*(2) Cell migration assay.* Cell migration was determined by the transwell assay and the wound healing assay. **(i) The transwell assay.** Cells grown in DMEM with 10% FBS were trypsinized and resuspended in DMEM with 10% FBS. 4 × 10^4^ cells were gently added to the upper compartment of Transwell (Corning). DMEM with 10% FBS or EGF were added to the lower compartment of Transwell. The cells were incubated in the culture incubator at 37°C plus 5% CO_2_ for indicated time. The remained cells on the upper side were gently removed with cotton balls. The cells migrated from the upper side to the lower side through the filter were fixed by 5% glutaraldehyde for 10 minutes, then stained with 1% Crystal Violet in 2% ethanol for 20 minutes. The stained cells on the lower side were counted under microscope from 5 different randomly selected views. The cell number averaged from the 5 microscopic views was used as the migration cell number. The migration experiments were repeated twice. **(ii) The wound-healing assay.** 8 × 10^5^ cells were seeded on 6-well plates in DMEM supplemented with 10% FBS. 16 hrs later, the cells reached to about 80-90% confluence in a monolayer. A pipette tip was used to make a straight scratch line in the cell monolayer. The cells were incubated for indicated times and treated as required. The experiments were repeated at least three times.

(3) *Cell invasion assay.* The transwell top chamber membrane was coated with 40 μl of 0.125 mg/ml matrigel in PBS and incubated at 37°C for 5 hrs before use. The gastric cancer AGS cells were trypsinized and washed with PBS 1–2 times and resuspended in DMEM at 5 × 10^5^ cells/ml. Before loading the cells, the matrigel layer was incubated in 300 μl of DMEM at 37°C for 30 min. The cells (200 μl in DMEM) were added to the top of the matrigel layer. The bottom chamber of the transwell was filled with 500 μl of DMEM plus 10% FBS. The cell invasion was carried out at 37°C and 5% CO_2_ for 24 hrs. After the invasion was done, the cells in the top chamber were removed by cotton balls. The cells on the bottom side (invaded cells) of the membrane were fixed with 4% paraformaldehyde at 25°C for 30 min and stained with 0.1% crystal violet solution at 25°C for 20 min. The stained cells were washed with PBS three times and observed and photographed under a phase microscope.

### Statistical analysis

The association between clinicopathological variables and Nedd4-1 status was tested using chi-square test. The outcome of interest was mortality. Kaplan-Meier survival analysis was conducted from post-surgery to death, stratified by Nedd4-1 expression status, TNM stage, T category, N category, tumor size, and differentiation, respectively. Log-rank test was used to test each of above factors. When analyzing association of Nedd4-1 expression with cumulative survival, TNM stage IV patients (33 cases) were excluded, because expression of Nedd4-1 in TNM stage IV GCA tumor samples has no prognostic value. The multivariate Cox proportional hazard model was used to determine the association between Nedd4-1 status and survival, controlling for tumor size, differentiation, T category, N category and TNM stage. Hazard ratio (HR) and 95% confidence interval (95% CI) were calculated for each factor. Interactions were also tested for each pair of factors. *P*-value <0.05 was considered to be statistically significant, and all analyses were conducted using IBM SPSS software (IBM Corp, Armonk, NY).

## Electronic supplementary material

Additional file 1: **Characterization of the anti-Nedd4-1 antibody.** (PDF 68 KB)

## Abbreviations

AEJ: Adenocarcinoma of esophagogastric junction
CI: Confidence interval
GCA: Gastric cardia adenocarcinoma
HECT: Homologous to the E6-AP Carboxyl Terminus
HR: Hazard ratio
HRP: Horseradish peroxidase.
