# Supplementary material for: Nedd4-1 is an exceptional prognostic biomarker for gastric cardia adenocarcinoma and functionally associated with metastasis
Source: Mol Cancer. 2014 Nov 14;13:248. doi: 10.1186/1476-4598-13-248 (PMC4239324; doi:10.1186/1476-4598-13-248)
Supplement: Supplementary file 1 — Additional file 1: Characterization of the anti-Nedd4-1 antibody. (PDF 68 KB) [file 12943_2014_1447_MOESM1_ESM.pdf]

Additional File 1: Characterization of the anti-Nedd4-1 antibody.

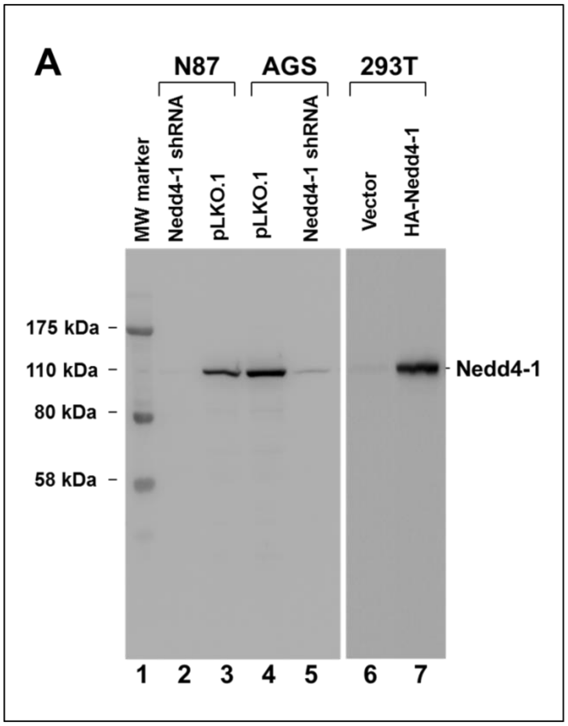

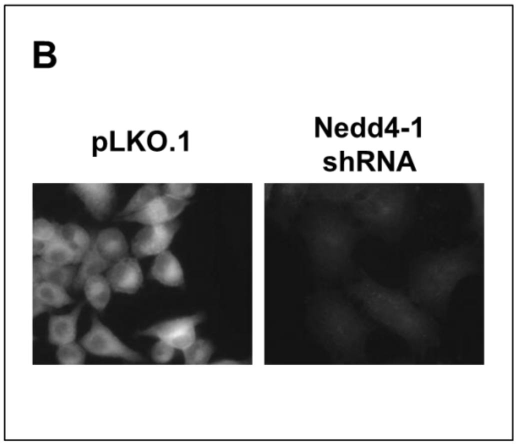

To test specificity of the anti-Nedd4-1 antibody, we first expressed HA-tagged Nedd4-1 in HEK293T cells. The control was transfected with the vector. As shown in Fig. A, the anti-Nedd4-1 antibody specifically recognized HA-Nedd4-1 in the lysates (lane 7). Notice that a weak band of endogenous Nedd4-1 was also detected in the vector control sample (lane 6). To further verify the specificity of the anti-Nedd4-1 antibody, we detected endogenous Nedd4-1 in gastric cancer AGS and N87 cell lysates with a Nedd4-1 knockdown (Nedd4-1 shRNA) cell lysate sample as a control. As shown in lanes 2-5 in Fig. A, the anti-Nedd4-1 antibody specifically recognized endogenous Nedd4-1 of the gastric cancer cells. The molecular weight markers are shown in lane 1.

To confirm that the anti-Nedd4-1 antibody recognizes native Nedd4-1 protein, we did immunofluorescent staining of AGS cells with or without knockdown of Nedd4-1. As shown in Fig. B, the anti-Nedd4-1 antibody stained the vector control cells, but not the Nedd4-1 shRNA-expressed cells, indicating that the anti-Nedd4-1 antibody recognizes native Nedd4-1 protein in AGS cells.
